# Impaired Cognitive Function after Perineuronal Net Degradation in the Medial Prefrontal Cortex

**DOI:** 10.1523/ENEURO.0253-18.2018

**Published:** 2018-12-28

**Authors:** John W. Paylor, Eszter Wendlandt, Tara S. Freeman, Quentin Greba, Wendie N. Marks, John G. Howland, Ian R. Winship

**Affiliations:** 1 Neurochemical Research Unit, Department of Psychiatry, University of Alberta, Edmonton, T6G 2R3 Canada; 2 Neuroscience and Mental Health Institute, University of Alberta, Edmonton, T6G 2E1 Canada; 3 Department of Physiology, University of Saskatchewan, Saskatoon, S7N 5E Canada

**Keywords:** cognition, memory, perineuronal nets

## Abstract

Perineuronal nets (PNNs) are highly organized components of the extracellular matrix that surround a subset of mature neurons in the CNS. These structures play a critical role in regulating neuronal plasticity, particularly during neurodevelopment. Consistent with this role, their presence is associated with functional and structural stability of the neurons they ensheath. A loss of PNNs in the prefrontal cortex (PFC) has been suggested to contribute to cognitive impairment in disorders such as schizophrenia. However, the direct consequences of PNN loss in medial PFC (mPFC) on cognition has not been demonstrated. Here, we examined behavior after disruption of PNNs in mPFC of Long–Evans rats following injection of the enzyme chondroitinase ABC (ChABC). Our data show that ChABC-treated animals were impaired on tests of object oddity perception. Performance in the cross-modal object recognition (CMOR) task was not significantly different for ChABC-treated rats, although ChABC-treated rats were not able to perform above chance levels whereas control rats were. ChABC-treated animals were not significantly different from controls on tests of prepulse inhibition (PPI), set-shifting (SS), reversal learning, or tactile and visual object recognition memory. Posthumous immunohistochemistry confirmed significantly reduced PNNs in mPFC due to ChABC treatment. Moreover, PNN density in the mPFC predicted performance on the oddity task, where higher PNN density was associated with better performance. These findings suggest that PNN loss within the mPFC impairs some aspects of object oddity perception and recognition and that PNNs contribute to cognitive function in young adulthood.

## Significance Statement

Perineuronal nets (PNNs) are organized components of the extracellular matrix that surround mature CNS neurons and are critical for the regulation of neuronal plasticity. A loss of PNNs has been observed in schizophrenia and other CNS diseases but the exact functional contribution of these structures or the consequences of their loss are not well understood. Here, we show that targeted degradation of PNNs within the medial prefrontal cortex (mPFC) disrupts performance of some tests of object oddity perception and recognition memory. These findings suggest that PNNs and their loss in CNS diseases may contribute directly to the presentation of cognitive dysfunction.

## Introduction

Perineuronal nets (PNNs) are highly organized components of the extracellular matrix that surround the cell body, proximal dendrites, and initial axon segment of mature CNS neurons (Hockfield and McKay, 1983; Wang and Fawcett, 2012). These structures play a critical role in the regulation of neuronal plasticity in the CNS (Pizzorusso et al., 2002; Sorg et al., 2016). PNNs act as a physical barrier to structural changes in the neurons and also stabilize the functional properties of these neurons. Consistent with this, PNNs are sparse early in development when plasticity is generally at its highest and increase throughout the postnatal lifespan, particularly following critical periods of plasticity (Pizzorusso et al., 2002; Mauney et al., 2013). Within these periods, cortical tissue undergoes dramatic structural reorganization of neural connectivity in response to the appropriate stimulus (Hensch, 2005). These changes are followed by a period of synaptic pruning, and then stabilization of the network long term. In line with a role in regulating plasticity, PNN expression increases at the closure of these critical periods and degradation of PNNs can re-open these windows of heightened plasticity in adulthood (Pizzorusso et al., 2002; Lensjø et al., 2017).

Several recent studies suggest that PNNs are reduced in the postmortem tissue of patients suffering from CNS disorders such as schizophrenia, epilepsy, and Alzheimer’s disease (Okamoto et al., 1994; Baig et al., 2005; McRae and Porter, 2012; Bitanihirwe and Woo, 2014; Pollock et al., 2014; Berretta et al., 2015). In schizophrenia, postmortem analyses of the prefrontal cortex (PFC), amygdala, and superior temporal cortex suggest reduced PNN density (Pantazopoulos et al., 2010; Mauney et al., 2013; Enwright et al., 2016). This finding has been replicated in animal models of the disease and coincides with the development of cognitive impairment (Paylor et al., 2016; Steullet et al., 2017). Postmortem analysis of Alzheimer’s patients has also revealed deficits in PNNs in the frontal lobe (Brückner et al., 1999; Baig et al., 2005; Morawski et al., 2010). Moreover, PNNs protect against Alzheimer’s pathology and their loss may render neurons particularly vulnerable to the disease pathology (Okamoto et al., 1994). PNN loss and the degradation of extracellular matrix components have also been implicated in epileptogenesis and the maintenance of seizures in epilepsy (McRae and Porter, 2012; Pollock et al., 2014). While this observational evidence is a compelling indicator that PNNs are involved in CNS disorders, our current understanding of their functional significance is limited. Studies that show coincidental PNN loss and behavioral disturbances are intriguing, but do not necessarily implicate the loss of PNNs as sufficient for causing cognitive dysfunction.

We have previously observed a reduction of PNNs in medial PFC (mPFC) of the offspring of rats exposed to polyI:C during pregnancy (Paylor et al., 2016). As an extension of these findings, the present study examined cognitive function after targeted reduction of PNNs in the mPFC of rats using chondroitinase ABC (ChABC). ChABC catalyzes the breakdown to glycosaminoglycan subunits of chondroitin sulfate proteoglycans (CSPGs), which are the primary component of PNNs (Brückner et al., 1998; Crespo et al., 2007). This treatment has been used extensively to degrade CSPGs in PNNs and the surrounding interstitial matrix (Fawcett, 2015). After injection, we assessed cognitive function using tasks where performance is impaired in the offspring of rats subjected to polyI:C during pregnancy, including altered object oddity preference, recognition memory, sensorimotor gating, and cognitive flexibility [set-shifting (SS) and reversal learning; Bissonette et al., 2013; Yang et al., 2014; Ballendine et al., 2015; Latif-Hernandez et al., 2016; Kamiński et al., 2017; Lins et al., 2018]. We found that ChABC treatment reduced overall extracellular matrix staining within the mPFC as well as a reduced density of PNNs. These cellular changes were associated with impaired performance on an object oddity task, and performance at chance levels in a task measuring cross-modal object recognition (CMOR). Interestingly, linear regression showed that PNN density predicted performance on the oddity task. Conversely, PNN digestion did not affect performance on measures of prepulse inhibition (PPI), SS, reversal learning, or tactile and visual object recognition memory. Thus, our findings support a nuanced effect of degrading mPFC PNNs on cognitive functions related to schizophrenia.

## Materials and Methods

### Subjects

Adult male Long–Evans rats (*n* = 80; 300–350 g; Charles River Laboratories) were used for all experiments. After their arrival, animals were pair housed in ventilated plastic cages and left undisturbed for 1 week with food and water *ad libitum* (Purina Rat Chow). A 12/12 h light/dark cycle was used with lights on at 7 A.M. Animals were given environmental enrichment in their home cage in the form of a plastic tube throughout the experiment. Following acclimatization, animals used for operant conditioning were maintained at 90% of free feeding weight and singly housed to ensure the appropriate amount of food was consumed by each rat in the home cage after behavioral testing. All animal procedures were performed in accordance with the University of Saskatchewan animal care committee’s regulations.

### Behavioral measures

All rats were handled for at least 5 min/d for 3 d before behavioral testing. They were also habituated to transport in an elevator from the vivarium to the testing rooms. Rats were randomly assigned to one of two groups for behavioral testing. Group 1 had ChABC or PEN infused into mPFC before testing two weeks later on PPI, the CMOR battery, and the oddity task. Group 2 was food restricted and then trained to press levers for food reward in the operant conditioning chambers. After passing SS train (see below), ChABC or PEN was infused into mPFC. Two weeks later, the rats were retrained on the SS train (3–4 d) and then tested on visual cue discrimination, SS, and reversal learning.

#### PPI

PPI measures the percentage attenuation of motor response to a startling tone when that tone is preceded by a brief prepulse. Two SR-LAB startle boxes (San Diego Instruments) were used. Each session had a constant background noise (70 dB) and began with 5 min of acclimatization, followed by six pulse-alone trials (120 dB, 40 ms). Pulse-alone (six trials), prepulse alone (18), prepulse + pulse (72), and no stimulus (six) trials were then presented in a pseudorandom order, followed by six additional pulse-alone trials. Prepulse + pulse trials began with a 20-ms prepulse of 3, 6, or 12 dB above background (70 dB). Prepulse–pulse intervals (time between the onset of the prepulse and the 120-dB pulse) were short (30 ms) or long (50, 80, or 140 ms). The intertrial interval varied randomly from 3 to 14 s (Howland et al., 2012; Lins et al., 2017).

#### CMOR battery

This task uses spontaneous exploratory behavior to assess visual memory, tactile memory, and visual-tactile sensory integration (Winters and Reid, 2010; Ballendine et al., 2015). The testing apparatus was a Y-shaped maze with one start arm and two object arms (10 × 27 cm) made of white corrugated plastic. A white plastic guillotine-style door separated the start arm from the object arms, and Velcro at the distal end of the object arms fixed objects in place. A removable, clear Plexiglas barrier could be inserted in front of the objects. A tripod positioned above the apparatus held a video camera that recorded the task activity. Rats were habituated to the apparatus twice for 10 min. Lighting alternated during habituation between white light (used during visual phases) and red light (used during tactile phases) for 5 min each with the order counterbalanced, and the clear barriers were in place for 1 d of habituation and removed for the other with order counterbalanced between all rats. Test days consisted of a 3-min sample phase with two identical copies of an object attached with Velcro to the maze, a 60-min delay, and then a 2-min test phase with a third copy of the original object and a novel object placed in the maze. Rats began each phase in the start arm; the guillotine door was opened and closed once the rat entered the object arms. This task consisted of three distinct tests performed on three separate days: tactile memory (day 1), visual memory (day 2), and cross-modal memory (day 3). Red light illuminated the tactile phases allowing the rats’ behavior to be recorded while preventing the rats’ visual assessment of the objects and the removal of the clear barriers allowed for tactile exploration. White light was used during visual phases, but clear Plexiglas barriers in front of the objects prevented tactile exploration. CMOR had a tactile sample phase (red light, no barriers) and a visual test phase (white light, clear barriers). Recognition memory was defined as significantly greater exploration of the novel object than the familiar object. Video recordings of behavior were manually scored by investigators blind to the treatment status of the rats and identity of the objects. Novel object preference was reported as a discrimination ratio (time exploring novel object – time exploring familiar object)/(total time exploring both objects) of the first minute of the test phase.

#### Oddity discrimination

The oddity discrimination test measures object perception using presentation of three copies of one object and a fourth distinct or “odd” object (Bartko et al., 2007). The testing apparatus was a square arena (60 × 60 × 60 cm) constructed of white corrugated plastic with Velcro in each of the four corners. Following 2 d of habituation to the arena (10-min sessions), the test day was conducted. On test day, three identical objects and one different or odd object made of glazed ceramic (a round “owl” statue, 9.5 cm in diameter × 8 cm tall) or plastic [a square Lego statue, 5.5 cm (w) × 7 cm (h)] were fixed to the Velcro and the rats’ activity were recorded for 5 min using a video camera mounted to the ceiling. The odd object and its location were counterbalanced among the rats in both treatment groups. Object exploration times were hand scored by an investigator blind to the treatment status of the rats. Object examination was counted when a rat’s face was oriented toward the object at a maximum distance of 2 cm. Odd object preference was reported as a percentage of the total time exploring the odd object. Note that 25% is chance performance in this task (Lins et al., 2018).

#### Operant SS task (OSST)

Eight operant conditioning chambers (MedAssociates Systems) in sound-attenuating cubicles were used. The chambers contained two retractable levers and two stimulus lights positioned on either side of a food port used to deliver food rewards (Dustless Precision Pellets, 45 mg, Rodent Purified Diet; BioServ). A 100-mA house light illuminated the chamber. Sessions began with levers retracted and the chamber in darkness (intertrial state), with the exception of lever training days in which the trial began with levers exposed to allow for baiting with ground reward pellets. Rats were tested once each day. For lever training, rats were trained to press the levers as described previously and immediately after reaching criterion, side preference was determined (Floresco et al., 2008; Thai et al., 2013; Zhang et al., 2012). For visual-cue discrimination, rats were trained to press the lever indicated by a stimulus light illuminated above it. Trials (every 20 s) began with an illumination of one stimulus light, followed 3 s later by the house light and insertion of both levers. A correct press of the lever underneath the illuminated stimulus light caused retraction of both levers and the delivery of a reward pellet. The house light remained illuminated for an additional 4 s before the chamber returned to the intertrial state. An incorrect press returned the chamber to the intertrial state (all lights off) with no reward. Failure to press a lever within 10 s of their initial insertion was scored as an omission and the immediate return of the chamber to the intertrial state. Strategy set-shift (shift to response discrimination), the visual-cue rule from the previous stage was reinforced with 20 trials where the rat was required to press the lever below the illuminated stimulus light. Subsequently, rats were required to change their response from the visual cue to a spatial cue (the lever opposite to their side preference, regardless of whether the stimulus light was illuminated) to receive a reward pellet. For reversal learning, rats were required to press the lever opposite to the one rewarded during SS. Criterion was 10 consecutive correct responses for each testing day and errors for each testing day were coded as described previously (Floresco et al., 2008; Zhang et al., 2012; Thai et al., 2013). Rats were tested for a minimum of 30 trials per day and a maximum of 150 trials per day. If a second day of testing was required, trials per criterion were calculated as the sum of the trials completed on all testing days for a given discrimination.

### mPFC infusions of ChABC or penicillinase (PEN)

Before and during the procedure, rats were anesthetized with the inhalant anesthetic isoflurane (Janssen). Preoperatively, all rats were administered a 0.5 mg/kg subcutaneous dose of the analgesic Anafen (Merial Canada Inc). After animals were positioned in the stereotaxic apparatus, the scalp was cut and retracted to expose the skull. Holes were drilled above mPFC and injectors made from 35Ga silica tubing (WPI) glued to PE-50 tubing were inserted bilaterally to the following coordinates: anteroposterior (AP) +3.0 mm; lateral (L) 0.7 mm; dorsoventral (DV) 4.4 mm relative to bregma. Either ChABC (100 units/ml) or PEN (100 units/ml) was infused (0.1 ul/min) for 2 min at DV coordinates –4.4 mm, –4.2 mm, and –3.9 mm (total infusion volume 0.6 μl/side). Injectors were left in place for an additional 6 min to allow for diffusion of the solution away from the last infusion site. Injectors were then slowly removed, the holes filled with bone wax, and wound was closed with stitches.

### Tissue collection

Following behavioral testing, rats were deeply anesthetized with isoflurane and transcardially perfused with PBS followed by 4% paraformaldehyde using infusion pumps. After perfusion, brains were extracted and stored in 4% paraformaldehyde at 4°C. One day later, brains were transferred to 30% sucrose for several days and then frozen in isopentane and optimal cutting temperature (OCT) gel. Frozen brains were sectioned at 25 µm on a cryostat. For cFos staining, animals (PEN = 8, ChABC = 8) were time-perfused 100 min after assessment on the oddity object task.

### Immunohistochemistry

Slides were warmed to room temperature for 20 min and then given three washes in 1× PBS for 10 min each. After which slides were incubated for 1 h with 10% Protein Block, serum-free (Dako) in 1× PBS. Slides were then incubated overnight at room temperature with a primary antibody in a solution of 1% Protein Block, 1% bovine serum albumin, and 99.9% 1× PBS with 0.1% Triton X-100. Primary antibodies were as follows: mouse anti-chondroitin-4-sulfate (C4S; 1:400; Millipore), *Wisteria floribunda* agglutinin (WFA; 1:1000; Vector Labs), mouse anti-parvalbumin (1:1000; Swant), rabbit anti-parvalbumin (1:1000; Swant); rabbit anti-IBA1 (1:200; Dako); mouse anti-GFAP (1:200; Sigma-Aldrich); c-Fos (1:400; Cell Signaling); mouse anti-GAD67 (1:400; Millipore); anti-gephyrin (1:500; ThermoFisher). After overnight incubation, slides were washed three times, twice in 1× PBS with 1% Tween 20 and once in 1× PBS. Slides were then incubated for 1 h with secondary antibodies in antibody solution (as above). Secondary antibodies were as follows: streptavidin 647 (1:200; Invitrogen), donkey anti-mouse Alexa Fluor 488 (1:200; Invitrogen), donkey anti-rabbit Alexa Fluor 647 (1:200; Invitrogen), and donkey anti-mouse 647 (1:200; Invitrogen). After 1 h of incubation, slides were washed again three times and mounted with 4’,6-diamidino-2-phenylindole (DAPI) in Vectashield mounting medium (Vector Labs).

### Microscopy

Images were acquired using a Leica DMI6000B Microscope with LAS AF computer software. The mPFC was identified using *The Rat Brain in Stereotaxic Coordinates* and selected based on landmarks in the DAPI nuclear staining pattern (Paxinos and Watson, 2007). The mPFC was identified between +2.76 and +3.24 mm anterior to bregma with the imaging window aligned to the midline and extending through cortical layers 1–6. All imaging was captured at 10× magnification with a total of six images taken bilaterally in adjacent sections (∼250 µm apart). Images from the primary somatosensory jaw (S1J) area were also taken from within the same slices (directly lateral) as images of the mPFC, as a control region outside of the targeted injection area. A constant gain, exposure, and light intensity was used across all animals. Gephyrin and neuronal nuclei (NeuN) confocal imaging was conducted on a LEICA SP5 confocal microscope. For each animal, four 2 × 2 tile scans were conducted at 25× magnification over the mPFC.

### Image analysis

Analysis was completed on unmodified images by an observer blind to the experimental condition of the tissue analyzed. Cell counts for DAPI+, IBA+, PV+, c-Fos+ cells, and gephyrin+ puncta were performed using the Image-based Tool for Counting Nuclei (Center for Bio-image Informatics, UC Santa Barbara, CA) plugin for NIH ImageJ software. PNNs were counted manually using ImageJ Cell Counter function. For cell specific gephyrin+ puncta, four cells were selected per image from each quadrant (total number of cells analyzed = 229). For PV+ immunofluorescence and GAD67 colocalization, an overlay for all PV+ cells was generated using the ImageJ Analyze Particles function and mean brightness values taken from both PV+ and GAD67+ channels within cell marked areas. A second analysis for PV+ and c-Fos+ cell density and colocalization was conducted using a custom automated detection script in Python (Python Software Foundation, Python Language Reference, version 2.7; http://www.python.org). For all images a standard rectangular area was drawn over the region of interest, spanning cortical layers 1–6, within which cells were identified and measurement parameters kept constant. For each stain measurements of mean brightness within the area were also taken. Quantification of densities are expressed as a 100 × 100 μm square (10 000 µm^2^).

### Statistical analyses

**Figure 1.**
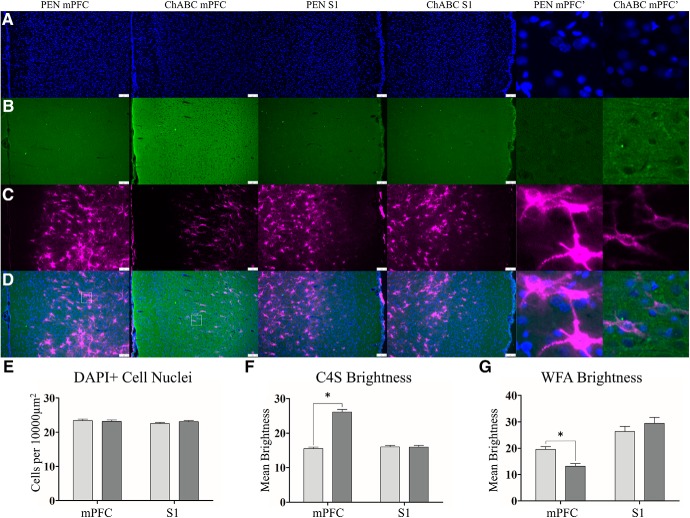
ChABC treatment increases C4S staining for cleaved CSPG stubs and decreases WFA expression of the extracellular matrix. Representative images of DAPI (***A***), C4S (***B***), WFA (***C***), and merges images (***D***). Within the mPFC, PEN-treated and ChABC-treated animals had no difference in total cellular density (***E***). PEN animals had minimal expression of C4S for cleaved CSPG stubs but after ChABC treatment this significantly increased (***F***). There was also a significant reduction in WFA expression in ChABC-treated animals (***G***). Similar analysis of the S1 (middle panels) of the same tissue slices from PEN-treated and ChABC-treated animals revealed no differences in C4S or WFA consistent with the localized injection and degradation we observed. Higher magnification images (right) images are 100 × 100 μm (10,000 μm^2^) insets taken from white-lined boxes (***D***, left). Scale bar (white): 100 μm. PEN, *n* = 40; ChABC, *n* = 40; *p < 0.05.

**Figure 2.**
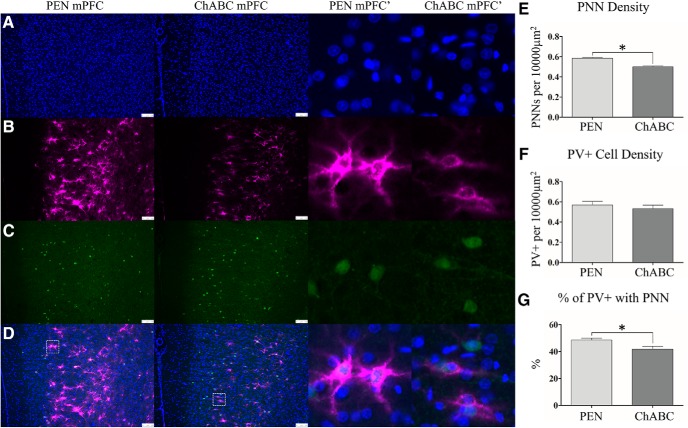
ChABC treatment reduced PNN density but did not affect PV+ interneurons. Representative images of DAPI (***A***), WFA (***B***), PV+ (***C***), and merges images (***D***). An examination of PNN density (***E***) showed that ChABC-treated animals had a significant reduction in PNNs. The density of PV+ interneurons was unchanged after PNN degradation (***F***). Higher magnification images (middle right) from the mPFC of PEN and ChABC showed that significantly less PV+ cells were surrounded by a PNN in ChABC-treated animals (***G***). Higher magnification images are 100 × 100 μm (10,000 μm^2^) insets taken from the white-lined boxes in (***D***). Scale bar (white): 100 μm. PEN, *n* = 40; ChABC, *n* = 40; *p < 0.05.

**Figure 3.**
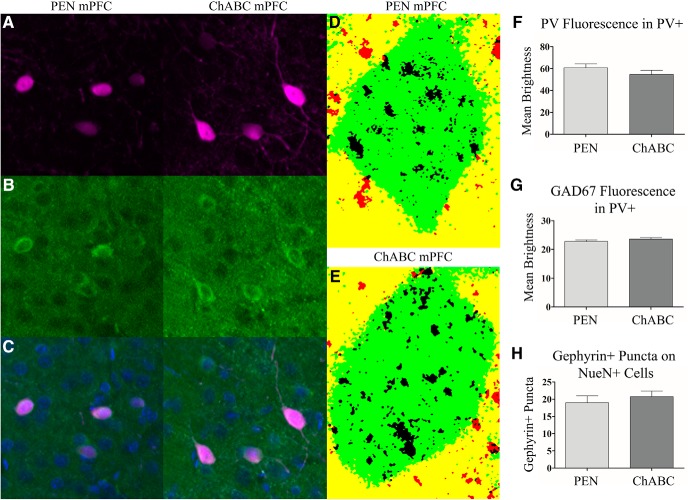
To evaluate the effect of ChABC treatment on PV+ cells (***A***), we examined PV+ and GAD67+ (***B***), cell fluorescence (merged in ***C***). Additionally, we examined the number of gephyrin+ puncta on neuronal cells labeled with NeuN (***D***, ***E***; NeuN+ cell = green, colocalized gephyrin+ puncta = black, puncta not colocalized with NeuN = red). ChABC treatment did not result in any change in PV+ fluorescence within PV+ cells (***F***). Similarly, GAD67+ expression in PV+ was not affected by ChABC (***G***). The number of gephyrin+ puncta colocalized with NeuN+ cells was also unaffected by ChABC treatment (***H***). Images are 100 × 100 μm (10,000 μm^2^) in size. PEN, *n* = 16; ChABC, *n* = 16.

**Figure 4.**
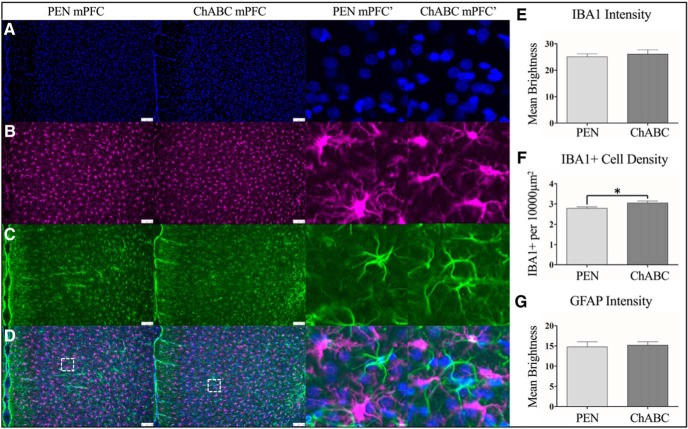
ChABC treatment increased microglial density but did not result in a robust immune response over PEN-treated control animals. Representative images are shown for DAPI (***A***), IBA1 (***B***), GFAP (***C***), and merged images (***D***). ChABC treatment did not result in overall changes in IBA1 staining intensity (***E***), but did cause a small but significant increase in IBA1+ cell density (***F***). Similar to IBA1, ChABC injection did not result in overt changes in GFAP staining intensity for astrocytes (***G***). Higher magnification images (middle right) are 100 × 100 μm (10,000 μm^2^) insets taken from the white-lined boxes in (***D***). Scale bar (white): 100 μm; *p < 0.05. PEN, *n* = 16; ChABC, *n* = 16; *p < 0.05.

**Figure 5.**
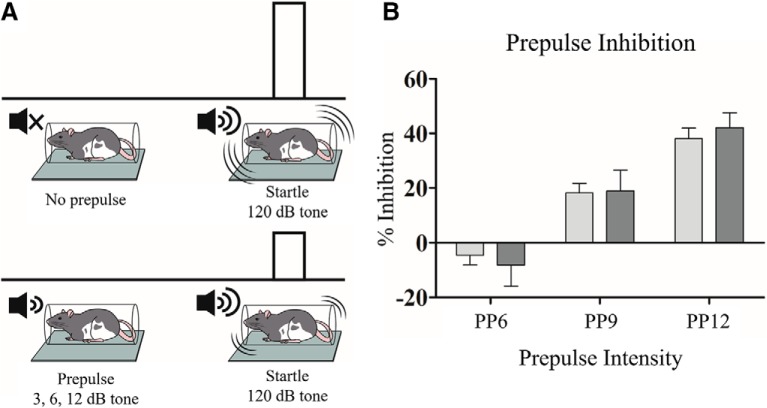
PNN degradation did not affect PPI. ***A***, Graphic representation of the behavioral assay. ***B***, Rats showed greater PPI for trials with increasingly loud prepulses. However, ChABC treatment did not affect PPI at any prepulse intensity. PEN, *n* = 25; ChABC, *n* = 24.

**Figure 6.**
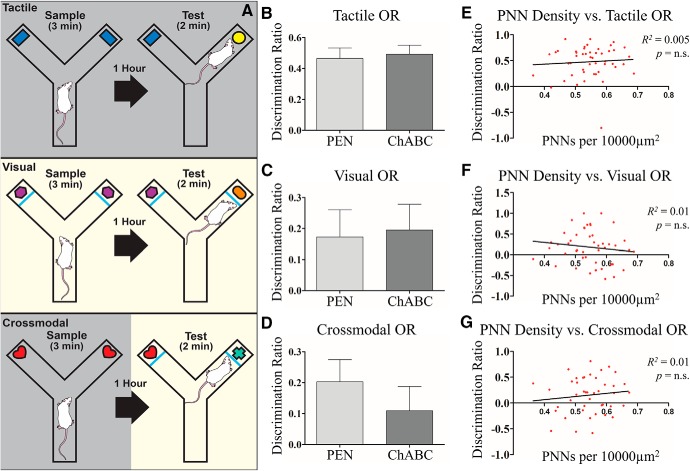
PNN degradation resulted impaired cross-modal recognition memory. ***A***, Graphic illustration of the behavioral assay. To emphasize the tactile modality (top) in object recognition, the lights are turned off during the task to limit rat’s ability to gather visual information about the object. In the visual phase (middle), the lights are on but the glass pane is positioned between the rat and the object, preventing them from gathering tactical information about the object. In the cross-modal phase (lower), animals are trained in one modality (e.g., tactile) and tested in the other (e.g., visual) to challenge integration across sensory modalities. ChABC treatment did not result in any changes in performance in tactile (***B***) or visual or (***C***) and after ChABC treatment, animals still performed significantly better than chance. In the cross-modal or (***D***) phase, animals treated with ChABC were not able to perform at better than chance levels whereas PEN-treated animals were. Linear regression was conducted to determine the predictive value of animals performance on the task of their PNN density, but no relationship was observed for the tactile (***E***), visual (***F***), or cross-modal (***G***) components of the task. PEN, *n* = 20; ChABC, *n* = 23.

**Figure 7.**
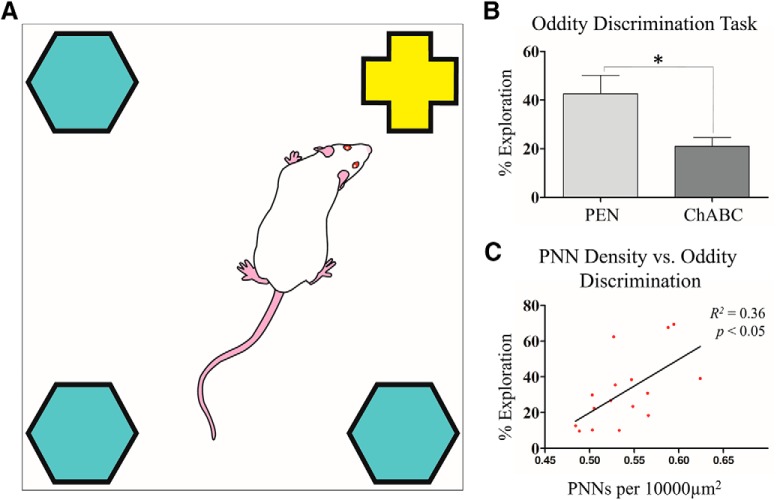
PNN degradation impaired performance on the oddity task and performance was predictive of PNN density. ***A***, Graphic illustration of the oddity task. Animals are presented with four objects, three of which are common and one of which is odd. ***B***, Animals treated with ChABC had a significant impairment in % exploration for the odd object compared to PEN animals. ***C***, Linear regression showed that animal’s PNN density, irrespective of treatment group, was predictive of performance on the oddity task. PEN, *n* = 8; ChABC, *n* = 8; *p < 0.05.

All data are presented as mean ± SEM. Statistical analyses were conducted in PRISM Software (Prism Software) and significance was set at *p* < 0.05. For experiments in Figures 1–3, 6, 7, unpaired Student’s *t* tests were used to compare PEN to ChABC. Simple linear regressions were used to examine the predictive value of behavioral performance on PNN densities. For Figure 4, a two-way ANOVA of treatment group and prepulse intensity was conducted to probe deficits in PPI. In Figure 5, in addition to unpaired Student’s *t* tests, we used one-sample *t* tests against chance performance to probe animals’ performance on object recognition. One-sample *t* tests to chance performance are frequently used in behavioral neuroscience to determine whether performance of a given group differs significantly from chance (Gervais et al., 2016; Jacklin et al., 2016; Lins et al., 2018).

## Results

### PNNs and interstitial matrix

To confirm the degradation of CSPGs and PNNs after treatment with ChABC, we stained with chondroitin-4-sulfate (C4S), a marker for cleaved components of CSPGs, and WFA, a marker for the CSPGs that preferentially labels PNNs (PEN = 40, ChABC = 40). Treatment with ChABC did not alter total cellular density (Fig. 1*E*) in the mPFC (*t*_(77)_ = 0.37, *p* = 0.72). Staining intensity for C4S was significantly greater in ChABC-treated animals than controls (*t*_(76)_ = 12.56, *p* < 0.0001; Fig. 1*F*). ChABC treatment induced a significant reduction in WFA staining intensity (*t*_(77)_ = 4.83, *p* < 0.0001; Fig. 1*G*) and a reduction in PNN density within the mPFC (*t*_(77)_ = 6.403, *p* < 0.0001; Fig. 2*E*). As a control to demonstrate selective digestion of PNNs at the site of injection, we assessed the same measures in the S1J, lateral from the mPFC, from within the same tissue slices. Within the S1J, total cellular density was not altered by ChABC treatment (*t*_(76)_ = 1.327, *p* = 0.19; Fig. 1*E*). C4S staining intensity (*t*_(76)_ = 0.07, *p* = 0.94; Fig. 1*F*) and WFA staining intensity (*t*_(76)_ = 1.03, *p* = 0.30; Fig. 1*G*) within the S1J were also unaffected by ChABC treatment. We also visually inspected slides anterior of the mPFC, including the frontal association cortex and regions of the orbitofrontal cortex and found no signs of elevated C4S or reduced WFA staining intensity. Similarly, there was no overt C4S or WFA alterations posterior in regions such as the hippocampus (data not shown).

### Parvalbumin-expressing (PV+) interneurons

PNNs most frequently surround PV+ inhibitory interneurons (Härtig et al., 1992). To assess whether changes in PNNs were paralleled by cellular loss of these inhibitory interneurons, immunostaining for an antibody specific to PV+ was performed (PEN = 40, ChABC = 40). Despite the close association between PNNs and PV+ inhibitory interneurons, the total density of PV+ cells was unchanged (*t*_(77)_ = 0.74, *p* = 0.46; Fig. 2*F*). However, the percentage of PV+ cells surrounded by a PNN was significantly reduced in ChABC-treated animals (*t*_(77)_ = 2.71, *p* < 0.01; Fig. 2*G*).

### GAD67 expression

To assess whether ChABC affected the integrity of PV+ cells, immunostaining for GAD67+, a critical GABA synthesis enzyme present in PV+ cells, was performed along with PV+ staining (PEN = 16, ChABC = 16). Across all images there was no difference between PEN and ChABC groups in terms of the number of cells analyzed (*t*_(29)_ = 1.28, *p* = 0.21). PV+ fluorescence within PV+ cells did not differ between groups (*t*_(29)_ = 1.17, *p* = 0.25; Fig. 3*F*). Similarly, ChABC treatment did not result in an overall change in GAD67+ fluorescence from within PV+ cells (*t*_(29)_ = 0.99, *p* = 0.33; Fig. 3*G*).

### Gephyrin+ puncta

To further examine the cellular consequences of ChABC treatment, we assessed gephyrin, a major scaffolding protein at inhibitory synapses, to determine whether PNN loss resulted in changes in inhibitory connectivity (PEN = 8, ChABC = 8). Within the mPFC, the total number of gephyrin+ puncta was not affected by ChABC treatment (*t*_(14)_ = 1.30, *p* = 0.22). Next, we assessed gephyrin+ puncta colocalized with NeuN, a marker for neuronal cells. A total of 229 cells were analyzed (average = 14.31 per animal) and measured cell size did not differ between PEN or ChABC animals (*t*_(14)_ = 0.27, *p* = 0.82). The number of gephyrin+ puncta colocalized with NeuN did not differ between groups (*t*_(14)_ = 0.67, *p* = 0.51; Fig. 3*H*)

### Immune cell labeling

To assess the degree of reactive inflammation to the injection of ChABC or PEN, immunostaining for IBA1+ microglia and GFAP+ astrocytes was performed (PEN = 16, ChABC = 16). Intensity of IBA1+ immunofluorescence was not altered by ChABC (*t*_(30)_ = 0.50, *p* = 0.61; Fig. 4*C*) but IBA1+ microglia cell density was significantly increased in treated animals (*t*_(30)_ = 2.31, *p* < 0.05; Fig. 4*D*). Treatment with ChABC did not significantly alter GFAP+ immunoreactivity (*t*_(30)_ = 0.28, *p* = 0.79; Fig. 4*E*).

### PPI

To assess whether PNN degradation resulted in deficits in sensorimotor gating, rats were tested on a PPI task using the presentation of acoustic stimuli. Rats showed a robust startle response to presentation of 120-dB tones in all treatment groups (PEN = 25, ChABC = 24). We observed a main effect of pulse block (*F*_(2141)_ = 56.65, *p* < 0.0001) indicating habituation of the startle response over the testing session. ChABC treatment resulted in a marginally increased startle response but this effect was not significant (*F*_(1141)_ = 3.20, *p* = 0.08). Rats in both treatment groups displayed greater PPI for trials with louder prepulses (Fig. 4*B*). A main effect of prepulse intensity (*F*_(2141)_ = 35.44, *p* < 0.0001) confirmed this observation (Fig. 4*B*). There was no main effect of treatment with ChABC on PPI (*F*_(1141)_ = 0.01, *p* = 0.93) and no interaction between prepulse intensity and treatment (*F*_(2141)_ = 0.25, *p* = 0.78). Linear regression was used to investigate the relationship between PNN density and PPI for 12-dB prepulses but no significant relationship was detected (*R*^2^ < 0.01, *p* = 0.91)

### CMOR

To assess whether PNN degradation affected recognition memory we assessed rats on a CMOR task (PEN = 20, ChABC = 23). Both groups showed similar levels of total object exploration during the sample phases of all three tests (tactile: PEN = 43.02 ± 2.44 s, ChABC = 47.57 ± 3.12 s; visual: PEN = 7.92 ± 0.68 s, ChABC = 7.92 ± 0.50 s; cross-modal: PEN = 46.14 ± 3.69 s, ChABC = 42.74 ± 3.12 s; statistics not shown). In the tactile object recognition testing phase, both groups had similar total exploration time of the objects (*t*_(46)_ = 1.31, *p* = 0.26; Fig. 6*B*) and discrimination ratio for the novel object was not affected by treatment (*t*_(46)_ = 0.32, *p* = 0.75). One-sample *t* tests revealed that rats in both groups displayed a preference for the novel object significantly greater than expected by chance (PEN *t*_(23)_ = 6.80, *p* < 0.001; ChABC *t*_(23)_ = 8.59, *p* < 0.001). In the visual object recognition testing phase, both groups had similar total exploration time of the objects (*t*_(46)_ = 0.21, *p* = 0.83; Fig. 6*C*) and discrimination ratio for the novel object was not affected by treatment (*t*_(46)_ = 0.19, *p* = 0.85). Rats in both groups displayed a preference for the novel object significantly greater than expected by chance (one-sample *t* tests, PEN *t*_(23)_ = 1.97, *p* = 0.03; ChABC *t*_(23)_ = 2.35, *p* = 0.01). In the CMOR testing phase, both groups had similar total exploration time of the objects (*t*_(41)_ = 1.54, *p* = 0.87). When comparing the discrimination ratio for the novel object, rats treated with ChABC were not significantly different from control rats (*t*_(41)_ = 0.86, *p* = 0.39; Fig. 6*D*). However, a comparison against chance showed that PEN rats performed significantly better than to be expected if rats had no recollection of the objects (one sample *t* test: *t*_(19)_ = 2.80, *p* = 0.01) whereas rats treated with ChABC did not perform significantly better than chance (*t*_(22)_ = 1.39, *p* = 0.09). Linear regression did not reveal significant relationships between PNN density and performance on visual, tactile, or cross modal object recognition (visual: *R* ^2^ = 0.02, *p* = 0.37; tactile: *R* ^2^ = 0.01, *p* = 0.62; CMOR: *R* ^2^ = 0.02, *p* = 0.44; Fig. 6*E–G*).

### Oddity task

As a second assessment of object recognition/perception, rats (PEN = 8, ChABC = 8) were tested on an oddity task to determine whether ChABC treatment impaired the ability to perceive and maintain representations of odd stimuli in their environment. There was no difference between total time exploring the objects for PEN or ChABC groups (*t*_(14)_ = 0.04, *p* = 0.96). When the percentage of exploration for the odd object was evaluated, ChABC-treated rats spent significantly less time inspecting the odd object compared to the duplicate objects than PEN-treated rats (*t*_(14)_ = 2.55, *p* < 0.05; Fig. 7*B*). Linear regression analysis identified a significant relationship between PNN density and the odd object preference in both groups (*R* ^2^ = 0.36, *p* < 0.05; Fig. 6*C*).

**Figure 8.**
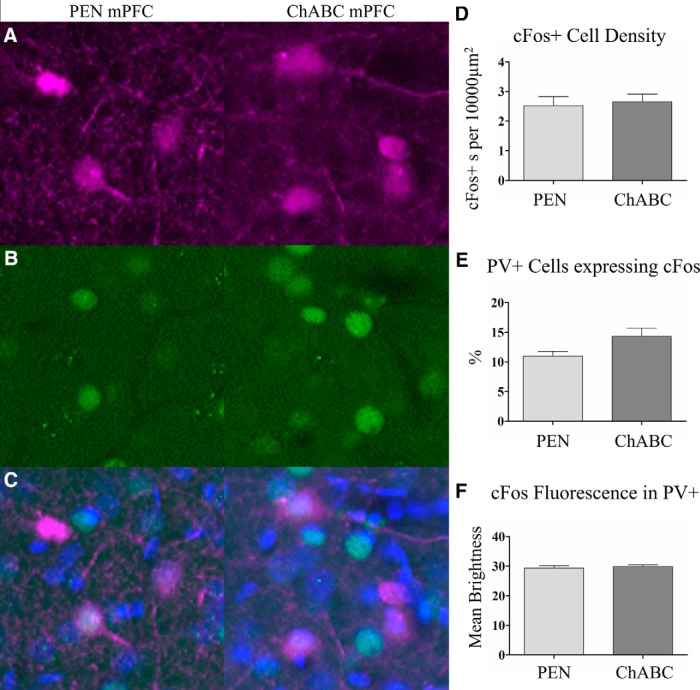
To evaluate the effect of behavioral testing on cellular activity, we time-perfused (100 min) a subset (*n* = 16) of animals after the oddity object experiment and examined c-Fos+ expression, a marker of heightened neuronal activity. Representative images for PV+ cells (***A***), c-Fos (***B***), and merged images (***C***). ChABC treatment did not result in a change in the total number of c-Fos+ cells within the mPFC, (***D***) it did however result in an slight increase in the number c-Fos+ colocalized with PV+, but this effect did not reach statistical significance (***E***). ChABC treatment did not affect c-Fos+ fluorescence within PV+ cells (***F***). Images are 100 × 100 μm (10,000 μm^2^). PEN, *n* = 8; ChABC, *n* = 8.

Rats evaluated on the oddity task were perfused 100 min after completion of the test to permit analysis of c-Fos immunoreactivity as a marker of neuronal activity in the mPFC (PEN = 8, ChABC = 8). Treatment with ChABC did not significantly alter the total number of c-Fos+ cells (*t*_(14)_ = 0.33, *p* = 0.75; Fig. 8*D*) nor was there a change in the intensity of c-Fos+ immunofluorescence in the cell soma (*t*_(14)_ = 0.56, *p* = 0.59; Fig. 8*F*). However, a comparison of the number of PV+ cells that colocalized with c-Fos+ immunoreactivity in ChABC animals relative to controls approached statistical significance (*t*_(14)_ = 2.10, *p* = 0.054; Fig. 8*E*).

### SS and reversal learning

To determine whether animals treated with ChABC had deficits in cognitive flexibility and learning, rats were assessed in SS and reversal learning paradigms. Rats in both groups (PEN, *n* = 15; ChABC, *n* = 16) had similar trials to reach criterion for the SS task (*t*_(29)_ = 0.16, *p* = 0.87) and a similar number of total errors (*t*_(29)_ = 0.16, *p* = 0.87). Comparison of perseverative errors only revealed no significant differences between treatment groups (*t*_(29)_ = 0.51, *p* = 0.61) nor did they differ statistically in regressive errors (*t*_(29)_ = 0.83, *p* = 0.42). A simple linear regression was used to determine the relationship between PNN density and total errors committed in the SS task but no relationship was found (*R*^2^ = 0.03, *p* = 0.34)

With regards to reversal learning, both PEN (*n* = 16) and ChABC (*n* = 16) rats required a similar number of trials to reach criterion (*t*_(29)_ = 0.34, *p* = 0.74) and committed a similar number of total errors (*t*_(29)_ = 0.04, *p* = 0.97). Errors committed by the two groups also did not differ when subdivided into perseverative errors (*t*_(29)_ = 0.57, *p* = 0.57) or regressive errors (*t*_(29)_ = 1.22, *p* = 0.23). A simple linear regression was used to determine the relationship between PNN density and total errors committed. There was a weak negative relationship between PNN density and total errors, but this effect was not significant (*R* ^2^ = 0.10, *p* = 0.08).

## Discussion

Here, targeted delivery of ChABC was used to degrade CSPGs and PNNs in the mPFC of adult rats. Immunohistochemistry confirmed that ChABC treatment elevated staining for C4S stubs, the cleaved disaccharide components of PNNs, and decreased WFA staining, a marker for CSPGs in the extracellular matrix. The density of PNNs was significantly decreased in mPFC by ChABC treatment. There was no change in the density of PV+ inhibitory interneurons, but the number of PV+ cells surrounded by a PNN was reduced. Furthermore, PV+ cells also had no change in the fluorescence of PV+ protein, c-Fos+, gephyrin, or GAD67. ChABC treatment significantly increased the density of IBA1+ microglia within the mPFC. Notably, PNN loss in the mPFC was accompanied by behavioral impairments in an oddity task and in CMOR, whereas PPI, SS, and reversal learning were unaffected.

### PNNs and cognitive function

The battery of tasks used in the present study was developed from previous research conducted to assess behavioral effects in the offspring of rats subjected to treatment with polyI:C during pregnancy. As the offspring of polyI:C-treated dams display altered behavior in these tasks (Howland et al., 2012; Zhang et al., 2012; Ballendine et al., 2015; Lins et al., 2018) and have reduced PNNs in mPFC (Paylor et al., 2016), we reasoned it would be valuable to assess behavior in the same tasks following ChABC infusions in young adulthood. In general, behavior of the PEN-treated rats was similar to that previously reported for these tasks (Ballendine et al., 2015; Marks et al., 2016; Lins et al., 2018); thus, we are confident in our testing protocols for these groups of rats. ChABC did not significantly affect PPI or alter the startle response. Although the mPFC is involved in the modulation of PPI in rats, an array of other brain areas are also involved (Swerdlow et al., 2001). Therefore, it is likely that the relatively subtle manipulation of mPFC PNNs we performed was insufficient to disturb the global activity of this circuit. Previously, deficits in frontal-dependent object recognition tasks, including object-in-place and CMOR, were observed in the male offspring of polyI:C treated dams (Howland et al., 2012; Ballendine et al., 2015). Other tasks, such as object recognition or the tactile and visual variants of the CMOR battery, were unaffected (Howland et al., 2012; Ballendine et al., 2015). Lesions of the orbitofrontal, but not mPFC, cortex impair performance of the CMOR task (Reid et al., 2014). As a result, it was somewhat unexpected that injections of ChABC into mPFC impaired performance of CMOR. Reconciling the effect of mPFC ChABC injections on CMOR with the lack of effect on the operant conditioning-based discrimination, SS, and reversal learning task battery is also difficult. In particular, temporary inactivation of the mPFC impairs the SS aspect of the task (Floresco et al., 2008). Thus, given the relatively subtle nature of the observed impairment of CMOR following mPFC ChABC injection, replication in future studies is important. The circuitry involved in the object oddity task is incompletely characterized, although no study to our knowledge has directly implicated the mPFC in this task. Previous work has shown the involvement of lateral cortical regions including perirhinal cortex in object oddity tasks (Bartko et al., 2007). As mPFC interactions with the perirhinal cortex are necessary for some object memory tasks (Hannesson, 2004), it is possible that interactions between these areas are also involved in the oddity task. However, this speculation will need to be tested directly.

These data contribute to a growing body of literature that suggests PNNs play an important role in cognitive function. PNN loss is associated with behavioral changes in several brain disorders (Pantazopoulos and Berretta, 2016), but relatively few studies have directly examined the effect of targeted PNN degradation on cognition. PNN degradation in the mPFC was recently shown to decrease the frequency of inhibitory currents onto mPFC pyramidal cells and impair cocaine-induced conditioned-place preference memory (Slaker et al., 2015). Consistent with our findings, PNN degradation was not associated with elevated network activity as indicated by the density of c-Fos+ cells, but the number of c-Fos+ cells ensheathed by a PNN was decreased. These findings differ from the trend toward elevated c-Fos+ expression in PV+ inhibitory interneurons observed in our data. Elevated c-Fos in PV+ neurons is consistent, however, with recent data showing ChABC treatment in the anterior cingulate cortex increased the fast rhythmic activity of GABAergic interneurons (Steullet et al., 2014). Interestingly, PNN degradation by genetic knock-out of the PNN component cartilage-link-1 protein or with ChABC treatment into the perirhinal cortex enhanced object recognition (Romberg et al., 2013). Similarly, genetic depletion of Tenascin-R, a PNN component, improved performance in reversal learning and working memory paradigms (Morellini et al., 2010). In contrast, genetic knock-out of Tenascin-C produced deficits in hippocampal-dependent contextual memory (Strekalova, 2002). These discrepancies may be explained by differences in the method and location of PNN manipulation, the memory task studied, and the time course of degradation and behavioral assessment. Memory impairment due to PNN disruption using ChABC depends on the timing of treatment in relation to memory formation. For example, removal of PNNs within the basolateral amygdala impairs conditioned fear memories but only if given before fear conditioning and extinction (Gogolla et al., 2009). Conversely, removal of PNNs within the basolateral amygdala impairs drug-associated memories, but only if given after memory formation but before extinction (Xue et al., 2014). Slaker et al. (2015) found that that WFA intensity after ChABC injection into the mPFC was reduced 3, 9, and 13 d following treatment but not at 30 d (Slaker et al., 2015), whereas PNN density was only significantly reduced 3 d postinjection and returned to control levels by 9 d. Conversely, our data shows that PNN density and WFA labeling intensity is still significantly reduced ∼25 d postinjection. These differences might be explained by animal strain differences (Sprague Dawley vs Long–Evans rats in our study) or injection volume (0.6 µl total volume vs 0.6 µl/side in our study) as ChABC concentration used were similar (0.09 units/µl vs 0.1 units/µl in our study).

### Functional consequences of PNN degradation

The effects of PNN degradation on neuronal structure and function are still poorly understood but can be considered in light of known PNN functions, including: (1) the regulation of GABAergic transmission, (2) restriction of neural plasticity, and (3) protection from oxidative stress and other environmental factors. PNNs are most frequently associated with PV+ fast-spiking GABAergic inhibitory interneurons. PV+ cells typically express the potassium channel KV3.1b, which is thought to give rise to their rapidly repolarizing action potentials. PNNs are thought to support these highly metabolically active neurons by acting as a buffers of excess cation changes in the local extracellular space (Härtig et al., 1999). The loss of PNNs has also been suggested to disrupt ion homeostasis and contribute to changes in functional activity of host neurons (e.g., hyperexcitability; Brückner et al., 1993). PNNs are important regulators of receptor function and localization on interneurons. During periods of elevated activity, synaptic glutamate AMPA receptors become desensitized and are exchanged for naïve receptors from the extrasynaptic pool (Heine et al., 2008). PNNs restrict this process, allowing for desensitization of synapses (Frischknecht et al., 2009). Degradation of PNNs might contribute to the hyper-excitability in neuronal cells that previously hosted PNNs. This is consistent with previous findings that ChABC treatment increases the firing rate of inhibitory interneurons (Dityatev et al., 2007). Our c-Fos immunolabeling did not conclusively identify increased immediate early gene activity in PV+ cells in ChABC-treated rats following the oddity task, but a comparison of the number of PV+ cells expressing c-Fos (relative to controls) approached statistical significance (*p* = 0.054).

PNNs also play a critical role in the regulation of neural plasticity, as evidenced by their role regulating critical periods of heightened plasticity during development (Takesian and Hensch, 2013; Sorg et al., 2016). Notably, PV upregulation denotes the onset of critical periods and the appearance of PNNs expression indicates the closure of critical periods (del Río et al., 1994; Hensch, 2005; McRae et al., 2007; Takesian and Hensch, 2013). In maturity, the degradation of PNNs can re-open critical periods of elevated structural and functional plasticity (Pizzorusso et al., 2002; Gogolla et al., 2009). Moreover, genetic knock-outs that disrupt PNNs (e.g., cartilage-link protein 1) can permanently delay the closure of the critical period and maintain a juvenile state of elevated plasticity well into adulthood (Carulli et al., 2010). Outside of critical periods, PNNs maintain similar plasticity-restricting properties. The degradation of PNNs with microinjections of ChABC enhances spine dynamics in hippocampal pyramidal cells (Orlando et al., 2012). Similarly, injections of ChABC into the visual cortex of adult mice can enhance spine dynamics and contribute to long-term functional synaptic plasticity (Pizzorusso et al., 2006; de Vivo et al., 2013). While digestion of PNNs in mPFC in our study was associated with varying degrees of impairment on cognitive tasks, we did not evaluate markers of neuroplasticity and it remains to be determined whether CSPG digestion induced aberrant neuroplasticity that contributed to these deficits.

Finally, PNNs may be protective against oxidative stress and other pathologic processes in CNS disease (Morawski et al., 2004; Suttkus et al., 2016). Fast-spiking PV+ interneurons are highly susceptible to oxidative stress and their association with PNNs is protective in immature and mature PV cells (Cabungcal et al., 2013; Suttkus et al., 2012). While it has not been directly demonstrated that PNN degradation in otherwise healthy animals results in oxidative stress injury, their loss may render neurons more susceptible to insult or disease. A recent study analyzed numerous genetic and environmental animal models of schizophrenia and identified oxidative stress in PV+ interneurons as a common feature in 12 of 14 models evaluated (Steullet et al., 2017). PNN loss was also present in 12 out of 14 of those models. While we did not detect overt loss of PV+ interneurons, increased oxidative stress in PV+ cells after PNN digestion could contribute to altered cognitive performance.

### PNNs in CNS disease

Our findings contribute to a growing body of literature that implicates PNNs and their loss in the symptomatology of CNS disorders such as schizophrenia, epilepsy, and Alzheimer’s (Okamoto et al., 1994; Baig et al., 2005; McRae and Porter, 2012; Bitanihirwe and Woo, 2014; Pollock et al., 2014; Berretta et al., 2015; Pantazopoulos and Berretta, 2016; Winship et al., 2018). Decreased PNN density in the PFC, superior temporal cortex, and amygdala has been reported in postmortem tissue from patients diagnosed with schizophrenia (Pantazopoulos et al., 2010; Mauney et al., 2013; Enwright et al., 2016). The loss of PNNs in the mPFC has also been recapitulated in animal models of schizophrenia (Paylor et al., 2016; Steullet et al., 2017). Our finding that PNN loss can disrupt performance on the CMOR task are of particular importance in this context, as polyI:C affected animals present with a CMOR deficit (Ballendine et al., 2015). In schizophrenia, disturbances to the inhibitory system have been reported, including loss of PV+ expression and GAD67, the GABA synthesis enzyme (Volk et al., 2000; Glausier et al., 2014; Kimoto et al., 2014; Enwright et al., 2016). CSPG digestions with ChABC did not induce significant changes in PV+ or GAD67+ fluorescence within PV+ cells. ChABC digestion induces a transient loss of CSPGs and PNNs, and it may be that altered PV and GAD67 expression in schizophrenia may results from chronic absence of PNNs around PV+ cells. Conversely, PNN decreases in schizophrenia may be the result of long-term, developmental dysregulation of PV+ cells which also disrupts the healthy expression of PV and GAD67. Similarly, we did not detect significant changes in the density of gephyrin+ puncta, which can be used to identify the presynaptic terminals of inhibitory synapses in the CNS. This suggests that our ChABC injections did not grossly modify the number of inhibitory synapses. However, our measurements are only sensitive to a net gain or loss of inhibitory synaptic contacts, and not changes to the turnover rate. Previous studies using *in vivo* imaging have shown that ChABC can destabilize dendritic spines and increase their motility while not affecting the net number, length, or volume (de Vivo et al., 2013).

## Conclusion

Our findings demonstrate that ChABC degrades PNNs and the interstitial matrix of the extracellular matrix in the mPFC. The loss of PNNs was associated with impairment in oddity object identification and object recognition memory. These findings contribute to growing body of literature suggesting that PNNs play an important role in healthy cognitive function and may have relevance for brain disorders (e.g., schizophrenia) where the pathology includes a loss of PNNs. While the mechanisms by which PNNs are reduced in these diseases is not well understood, interventions that target the loss of PNNs or stimulate their development could reduce cognitive impairment in neurodevelopmental or neurodegenerative diseases.

## Synthesis

Reviewing Editor: Tim Bussey, Western University Decisions are customarily a result of the Reviewing Editor and the peer reviewers coming together and discussing their recommendations until a consensus is reached. When revisions are invited, a fact-based synthesis statement explaining their decision and outlining what is needed to prepare a revision will be listed below. The following reviewer(s) agreed to reveal their identity: Boyer Winters.

Hi Ian,

A few words to explain the decision. I've now received two reviews on your MS. Both reviewers express a certain amount of enthusiasm for the study, but both have some quite serious concerns.

Reviewer 1 is primarily concerned with the behaviour, and is concerned that there is little rationale for the tasks chosen. Even more serious, perhaps, is their concern that some of the tasks used may not be PFC-dependent. Although the reviewer's evaluation of the MS was quite negative, I'd like to give you the opportunity to respond to these concerns and explain the putative disconnects in a revision of the MS, as it is possible you may be able to provide additional information or qualifications such that the rationale and results of the behaviour can make sense in the context of previous literature.

Reviewer 2 has more serious concerns regarding the histological analysis. Not to put too fine a point on it, they feel it is of insufficient quality to be published. Not being strongly expert in this area myself, I took the liberty of consulting another colleague who gave me their informal assessment. I'm afraid their assessment was the same as that of Reviewer 2, commenting in particular that the magnifications used are not sufficient to convincingly visualise PNNs. (For your information they also made some additional comments in passing, such as why when counts are expressed per square 1000um the figures show not a square but a rectangle, what is the IEG cfos showing when the analysis is not immediate, and they questioned the brightness of C4S as a measure of PNNs and wondered why is it still present at the time of analysis and not already metabolised.)

At eNeuro we try not to ask authors for a load of additional experiments, to avoid the unpleasant situation whereby things don't work and the study remains unpublishable (we've all been there). So the question for me is whether re-doing the histology constitutes more experiments (&gt;say, 2 months work). I'm going to assume that it doesn't, and that this could be done in a revision. So I'm rendering a decision of Major Revisions. If you think the work can't be completed in that time frame I understand. In any case, we really appreciate your submitting to eNeuro, and hope it works out.

Reviewers comments in full below

All the best

Tim

Reviewer 1

The contribution of PNNs to cognitive function is only just now starting to be probed extensively, and many questions remain. This study adds to a growing literature implicating PNNs in aspects of cognition.

The manuscript presents a study evaluating the necessity of perineuronal nets (PNNs) for cognitive functions in the rat. The authors used the enzyme chondroitinase ABC (ChABC) to degrade PNNs in the medial prefrontal cortex (mPFC) of rats prior to testing on a variety of cognitive tests, including crossmodal object recognition (CMOR), object oddity, set-shifting, reversal learning, and pre-pulse inhibition. Only the object-based tests were impaired by ChABC administration, and higher PNN density in mPFC was correlated with better performance on the oddity task. The authors conclude that PNN loss within the mPFC impairs “recognition memory” and that these results may have implications for understanding human cognitive disorders characterized by changes to PNN.

The procedures are quite clearly described and appear to be sound. The authors present a relatively thorough characterization of the effects of ChABC in the mPFC, and behavioural results are clear, if not necessarily adequately discussed.

I have several comments/questions for clarification.

1. The rationale for studying PNN contributions to memory is well established in the Introduction.

2. However, the same cannot be said for the choice of behavioural tests. While they do refer to models of schizophrenia and Alzheimer's disease, the authors do not clearly establish good reasons for some of the tasks used. The studies cited on p. 4 refer to working memory, set-shifting, and reversal tasks. There is actually little rationale provided for the choice of object-based tests, an important point considering these are the tasks on which effects were seen. Indeed, throughout the manuscript, there is a somewhat glossing-over of the nature of these tasks and the reason for their use. In the abstract, the authors refer to the effects as impairing “recognition memory”, but clearly basic recognition memory is not affected here, as performance on the CMOR control tasks (visual and tactile) is intact in ChABC-treated rats. I think a slightly more nuanced approach to the introduction and discussion of these tasks and effects is probably warranted. Jacob Cloke, Derek Jacklin and colleagues published a handful of studies showing effects in schizophrenia models on the CMOR task; citing these could help to establish relevance.

3. In a similar vein, the authors state throughout that these are mPFC-dependent tasks, but I am not aware of studies demonstrating that object oddity (typically referred to as a perceptual - not mnemonic - task), relies on mPFC. This is not to say that it does not, but could the authors provide a basis from the literature for this statement? Similarly, and perhaps more troubling for their rationale, the authors state that the CMOR task is mPFC-dependent, but I believe Reid and colleagues published a paper looking at PFC contributions and showed no effect of bilateral mPFC lesions in rats.

4. Closely related to that last point, as well as being more broadly relevant: The authors present no data regarding possible effects of ChABC BEYOND mPFC. Did they analyse PNN density, etc. in other brain regions, either as a control measure or out of curiosity? If so, these should be included. Is it possible that ChABC also affected orbitofrontal cortex (which I believe the Reid et al paper actually implicated in CMOR task performance)?

5. Several procedural details appear to be missing for the set-shifting task. How many trials were run per day for each stage of training/testing? What were the specific acquisition criteria for these stages? ITI length?

6. When describing results from the oddity task on p. 15, authors refer to the ‘novel’ and ‘familiar’ objects. Better terminology might be ‘odd’ and ‘repeated’ or ‘common’ (as used in the Fig 6 caption). Technically, all objects should be equally novel in this task, at least at the start of the trial.

7. As implied above, the discussion would benefit from a more nuanced take on the selectivity of behavioural effects reported. How do the authors' proposed mechanism of PNN contribution relate to the fact - if at all - that only the object-based tasks were impaired by ChABC?

Reviewer 2

The authors present intriguing results. But the approach to manipulate PNNs is not novel, and probably another model can be required. The behavioural data seemed to support the hypothesis but the methodology and the analysis is not convincing. Thus, iit is difficult to gauge the validity of the data. The manuscripts falls short of identifying a mechanism between losing PNNs and and animal data.

Statistics:

The authors provided only total number of animals used for all the experiment. The numbers of groups for each experiment and the analysis of IHC are required for validation of the statistics. The description of statistics in Methods should provide more details. The statistical significance of cross-modal object recognition test (Fig5,F) is not agreeable since it is not a group comparison. The data is not significant when ChABC group is compared to PEN group as the authors mentioned in the manuscript. This result authors claims as one of the cognitive deficits is not statistically significant.

There is a moderate level of enthusiasm. however there are some major concerns.

1. This manuscript could have been written more organised manner. Scientific discussion or speculations for the results are not thorough enough to convince.

2.The IHC data was not presented in a precise manner so it is rather confusing. The proper units for Y axis are all missed in the figures. The representative images of PV (Fig 2, Fig 7) are not convincing. Moreover it doesn't show any evidence to suggest mechanism between PV/cfos and PNNs digestions in the mPFC. The quantification of PV+ cells may not be affected by ChABC but there is no speculations suggested by authors in order to explain the link between PV, ChABC and cognitive deficit. It is also recommended that authors should provide the intensity fractions of PV accumulation (e.g.High vs Low intensity) rather than the cell density. Moreover, levels of inhibitory synaptic molecules (e.g.gephyrin) should be assessed after treatment too.

3. The behavioural tests used in the study seemed to be not well validated. Authors did not mention how they calculated the discrimination ratio. Visual OR test (Fig 5,E) shouldn't be valid since the discrimination ratio is closed enough to chance level considering the big error bars. PEN treated group should score between at least 0.2 - 0.4. In the oddity task authors should provide more details of experiments in terms of object they used. How many objects pairs were used?Was it counterbalanced or only one set-up? This is important to provide how authors planned behavioural experiments in order to avoid any bias.

4.All the N numbers are missing for each experiment throughout the manuscript. The statistical analysis of CMOR was not scientifically acceptable. One sample t-test to chance ?? And authors shouldn't say bluntly that they detected a statistical trend as written in Pg14. it seems to be a trend but it is still not significant and it may be improved by adding more N number if required.
